# Sensitivity to intrinsic rewards is domain general and related to mental health

**DOI:** 10.1038/s44220-023-00116-x

**Published:** 2023-09-06

**Authors:** Bastien Blain, India Pinhorn, Tali Sharot

**Affiliations:** 1 Affective Brain Lab, Department of Experimental Psychology, University College London, London, UK; 2 Max Planck UCL Centre for Computational Psychiatry and Ageing Research, University College London, London, UK; 3 Centre d’Economie de la Sorbonne, Paris 1 Panthéon-Sorbonne, Paris, France; 4 Department of Brain and Cognitive Sciences, Massachusetts Institute of Technology, Cambridge, MA USA

**Keywords:** Psychology, Motivation

## Abstract

Humans frequently engage in intrinsically rewarding activities (for example, consuming art, reading). Despite such activities seeming diverse, we show that sensitivity to intrinsic rewards is domain general and associated with mental health. In this cross-sectional study, participants online (*N* = 483) were presented with putative visual, cognitive and social intrinsic rewards as well as monetary rewards and neutral stimuli. All rewards elicited positive feelings (were ‘liked’), generated consummatory behaviour (were ‘wanted’) and increased the likelihood of the action leading to them (were ‘reinforcing’). Factor analysis revealed that ~40% of response variance across stimuli was explained by a general sensitivity to all rewards, but not to neutral stimuli. Affective aspects of mental health were associated with sensitivity to intrinsic, but not monetary, rewards. These results may help explain thriving and suffering: individuals with high reward sensitivity will engage in a variety of intrinsically rewarding activities, eventually finding those they excel at, whereas low sensitivity individuals will not.

## Abstract (web summary)

In this cross-sectional study, Blain and colleagues show that sensitivity to diverse visual, cognitive and social, putatively intrinsic rewarding stimuli is partly domain general and is linked to affective aspects of mental health unlike sensitivity to monetary rewards.

## Main

Humans spend much of their time engaging in activities that are pleasurable in their own right. These activities are undertaken even when they do not lead to external outcomes: they are intrinsically rewarding. Watching the sunset, reading, solving crossword puzzles, playing, exploring nature and observing works of art are a few such examples.

On the surface these different activities do not have common features or goals. This contrasts with activities that lead to primary rewards (for example, eating, fornicating), which all have clear and direct survival benefits, and secondary rewards (for example, money), which in turn are associated with primary rewards. It is possible, however, that different intrinsic rewards do share core characteristics, mechanisms and goals not readily transparent^1,2^. If so, such common features should elicit similar types of behavioural responses, and individual differences in these responses should be partially domain general. We test this hypothesis in this work, namely, that despite diverse intrinsic rewards seeming vastly different from each other, sensitivity to them is partially domain general and may be shared with secondary rewards.

Engagement with specific intrinsically rewarding stimuli has been associated with happiness^3,4^, mental health^5^ and professional achievement^6^. Here we pose that these past findings can in fact be explained by a core association between mental health and domain-general sensitivity to intrinsic rewards. That is, if an individual finds a specific stimulus rewarding (for example, observing landscapes), they may be more likely to find other stimuli (reading, playing and so on) rewarding due to a domain-general sensitivity to (intrinsic) rewards, which may be associated with mental health. Individuals with high sensitivity to intrinsic rewards will be inclined to engage with a variety of seemingly diverse intrinsically rewarding activities, which in turn will increase the likelihood that they will eventually find rewarding activities that they also excel at. Low sensitivity to intrinsic rewards, on the other hand, will produce a general disinterest in a large variety of activities, which will lead to low mood and lack of motivation. Thus, a domain-general sensitivity to intrinsic rewards will contribute to flourishing and its absence to suffering.

**Fig. 1:**
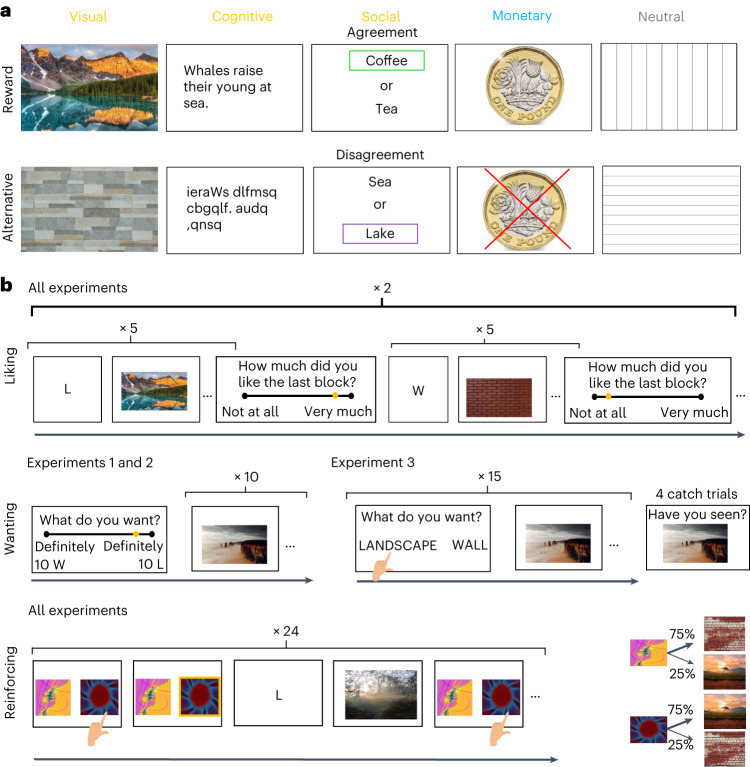
Experimental design. **a**, Stimuli included five categories: visual, cognitive, social, monetary rewards and neutral stimuli. Intrinsic rewards were the visual reward (landscapes), the cognitive reward (facts) and the social reward (social approbation of participant’s preference, which was collected at the beginning of the experiment). The monetary reward was a coin signalling a bonus payment. It was always the same coin in experiments 1 and 2, but different coins on every trial in experiment 3. Each reward was associated with an alternative stimulus. This included a wall for the visual reward, a random string of letters for the cognitive reward, disagreement for the social reward, and not receiving a coin for the secondary reward. Neutral stimuli were vertical and horizontal lines. **b**, For each reward type, participants were exposed to two blocks of the rewarding stimulus (five trials each) and two blocks of the alternative stimulus (five trials each). Order was reward–alternative–reward–alternative or alternative–reward–alternative–reward. After each block, participants rated how much they liked that block. To measure wanting in experiments 1 and 2, participants were asked to rate how much they wanted to be exposed to ten trials of the rewarding stimulus or ten trials of the alternative stimulus. They were then exposed to ten trials of the chosen stimulus. In experiment 3, on each of 15 trials, participants chose whether to be exposed to a rewarding stimulus or the alternative stimuli, and then their choice was honoured. To measure the reinforcing strength of each reward, participants were exposed to a pair of abstract cues each probabilistically related to the rewarding stimulus with either 0.75 probability or 0.25 probability and to the alternative stimulus with either 0.25 or 0.75 probability. The percentage of trials in which they selected the abstract cue leading more often to the rewarding stimulus was the measure of the reinforcing strength. Each reward type was presented in counterbalanced order across participants for all experiments. In experiments 1 and 2, the order of measures was liking–wanting–reinforcement, whereas in experiment 3, it was counterbalanced across participants and reward types. In this figure we use landscapes (L) and walls (W) as example trials.

We focus here on a core aspect of mental health that we will refer to as affective health. We define affective health as a range of characteristics that are related to positive mood, high motivation, feelings of pleasure, interest and happiness. To investigate whether sensitivity to intrinsic rewards is domain general and related to affective aspects of mental health, we selected three putative intrinsically rewarding stimuli from the visual^7,8^, cognitive^9,10^ and social^11,12^ domains (Fig. 1a). We also compared the responses to these putative intrinsic rewards with responses to monetary rewards to test whether sensitivity to intrinsic rewards is shared with that to secondary rewards. Note that by using the term intrinsically rewarding, we do not refer to intrinsic motivation or internally motivated activities (terms often used to describe activities associated with self-generated goals), but rather to stimuli and activities that are enjoyable even in the absence of a clear goal.

It has been suggested that a stimulus is a reward if it elicits three typical responses^13^. First, it elicits positive emotions (it is ‘liked’). Second, it generates approach/consummatory behaviours (it is ‘wanted’). Third, it increases the likelihood of the action that led to it (it is ‘reinforcing’). We therefore measured liking, wanting and reinforcement of all stimuli to assess individuals’ reward sensitivity. We also asked participants to fill a range of questionnaires related to affective aspects of mental health and implemented a dimensionality approach^14–16^, which considers the possibility that specific symptoms are predictive of several conditions, thus allowing an investigation that cuts through classic clinical boundaries. Together, the data allowed us to examine if within-individual responses to intrinsic rewards are domain general and linked to mental health.

## Results

Two identical online studies were conducted (experiment 1, *N* = 132; experiment 2, *N* = 171), as well as a modified version (experiment 3, *N* = 180; see the flow chart in Supplementary Fig. 1). We measured three types of responses in each study: liking, wanting and reinforcing strength (in that order in experiments 1 and 2, and counterbalanced in experiment 3; see Methods) to ten categories of stimuli. There were three categories of putative intrinsic reward (visual, cognitive, social) and three categories of non-rewarding alternative stimuli (visual, cognitive, social). In the visual domain, we used landscapes as a reward and images of walls as an alternative (Fig. 1a and Methods). Staring at landscapes has been rated positively in the past unlike looking at walls^7,17^. In the cognitive domain, we presented participants with trivia facts, as consuming such information (for example, reading) is thought to be rewarding^9,10^. It has been shown that humans select to observe (and thus read) sentences and that the opportunity to consume sentences that increase general knowledge activates the reward system just like primary rewards, suggesting that consuming knowledge is rewarding^9,10^. We presented a random string of letters as an alternative. In the social domain, we used social similarity as a reward (that is, a participant learns that another participant shares the same preference as them) and social disagreement as an alternative. This was selected as studies have exhibited the existence of a confirmation bias by which subjects select to observe information that they suspect confirms their beliefs (for example, ref. ^18^), including in the social domain^11^. Moreover, confirmation has been shown to activate the reward system^11^, suggesting that consuming confirmatory information (for example, learning that someone agrees with you) is rewarding. The monetary reward was earning bonus money (represented on screen as a coin) and its alternative was not receiving a coin. All reward types were compared with a neutral stimulus: vertical and horizontal lines.

To measure liking, participants were exposed to a block of a rewarding stimulus and to a block of its alternative, and were asked to report how much they liked that block after each block (Fig. 1b and Methods). To measure wanting in experiments 1 and 2, participants indicated whether they preferred to experience ten trials of the reward or ten trials of its control on a sliding preference scale, which deterministically and explicitly led to the presentation of either ten trials of the putative intrinsically rewarding stimulus or its alternative. In experiment 3, on each of 15 trials, participants chose whether to be exposed to a rewarding stimulus or to its alternative and their choice was immediately honoured. To measure reinforcing strength, that is, whether a stimulus increases the likelihood of the action that preceded it, participants chose between abstract shapes probabilistically linked to the putative intrinsically rewarding stimulus or to the corresponding alternative.

### Intrinsic rewards are liked, wanted and reinforcing

**Fig. 2:**
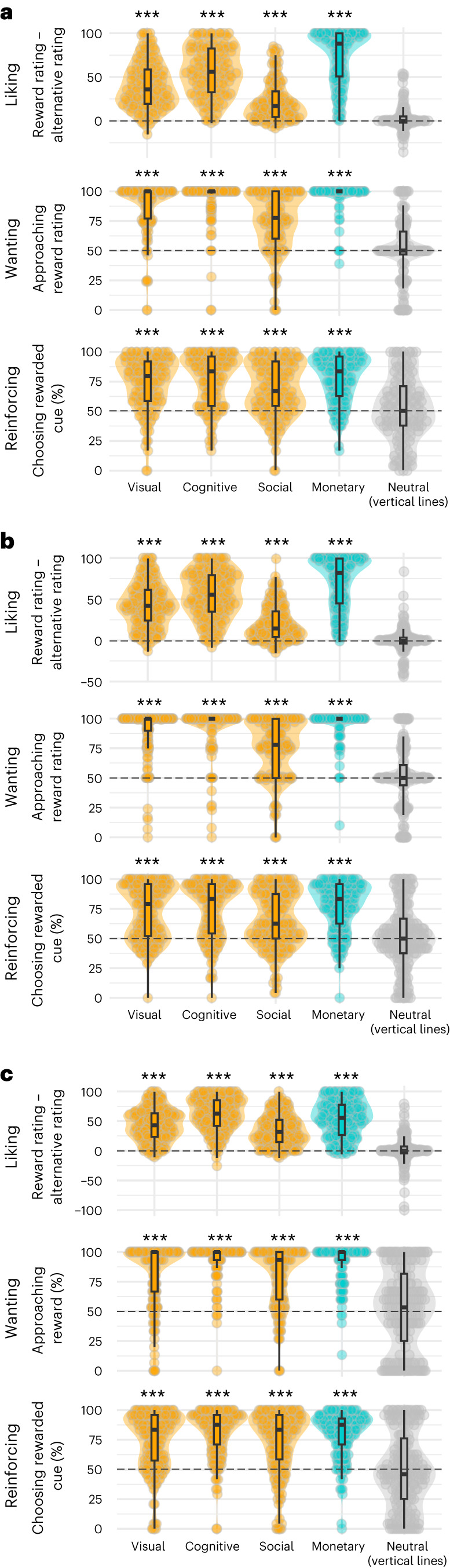
Putative intrinsic rewards are liked, wanted and reinforcing. **a**–**c**, Intrinsic rewards elicit the same type of responses as monetary rewards, unlike neutral stimuli, in experiments 1 (**a**, *N* = 132 participants), 2 (**b**, *N* = 171 participants) and 3 (**c**, *N* = 180 participants). Each row corresponds to a behavioural response: liking, the difference in average liking of the rewarding block versus the alternative block; wanting, the average rating of wanting to experience the intrinsically rewarding block versus the alternative block (50 is indifference) in experiments 1 and 2, and the proportion of reward choice in experiment 3; reinforcing, the proportion of trials in which the cue leading more often (75%) to the reward was selected (chance level is 50%). Each column corresponds to a stimulus type. The shaded areas show the probability density of the data at different values, smoothed by a kernel density estimator. Each dot represents a participant. The box plots show five summary statistics (bold horizontal lines represent the median; the two hinges correspond to the first and third quartiles; and the two whiskers correspond to 1.5 times the interquartile range). The dashed horizontal black lines correspond to the chance level. Two-sided Wilcoxon signed rank tests (non-parametric). ***P < 0.001. *P*-values are reported in Supplementary Table 1.

We first examined how participants responded to monetary rewards (Fig. 2a–c, blue data, and Supplementary Table 1). As expected, monetary rewards were rated higher on the liking scale (from 0 (disliked a lot) to 100 (liked a lot)) than their absence and rated higher than indifference on the wanting scale (experiments 1 and 2), and selected more often than the alternative (experiment 3). The cue leading to monetary reward was chosen more often than chance (see Supplementary Table 1 for all statistics).

We next examined whether intrinsic rewards also elicited liking, wanting and reinforcement. These stimuli were indeed liked more than their alternative, as well as more than any other alternative (see Supplementary Table 2 for all statistics). All intrinsic rewards were also rated higher on the wanting scale than indifference (experiments 1 and 2) and selected more often than their alternative (experiment 3). Finally, the likelihood of selecting the cue leading to the putative intrinsic reward was greater than chance (see Supplementary Table 1 for all statistics). Together, these results suggest that these stimuli were (intrinsically) rewarding and revealed that they trigger the same type of qualitative responses as to monetary rewards (Fig. 2a–c, orange data). This does not mean that the average magnitude of responses is similar across reward categories, but rather that a similar type of responses is observed.

By contrast, the neutral stimuli (vertical and horizontal lines) did not elicit the same type of responses as monetary and intrinsic rewards. Vertical lines were not more liked than the horizontal lines in experiments 1, 2 and 3 (Supplementary Table 1), and lines were less liked than any other rewards (Supplementary Table 3). Neither vertical nor horizontal lines were rated on the wanting scale above indifference (experiments 1 and 2) or were chosen more than chance (experiment 3; see Supplementary Table 1). Nor were they reinforcing, that is, the cue leading to vertical lines was not selected above chance (Supplementary Table 1).

So far, the results revealed that the putative intrinsically rewarding stimuli and monetary rewards show the three behavioural signatures of reward: they are liked, wanted and reinforcing, whereas neutral stimuli are not.

### Reward sensitivity is partially domain general

We next tested whether the sensitivity to intrinsic reward is domain general. In other words, if a particular participant finds the visual reward rewarding, will they be more likely to find the cognitive and the social reward rewarding? Will they also find the monetary reward rewarding?

**Fig. 3:**
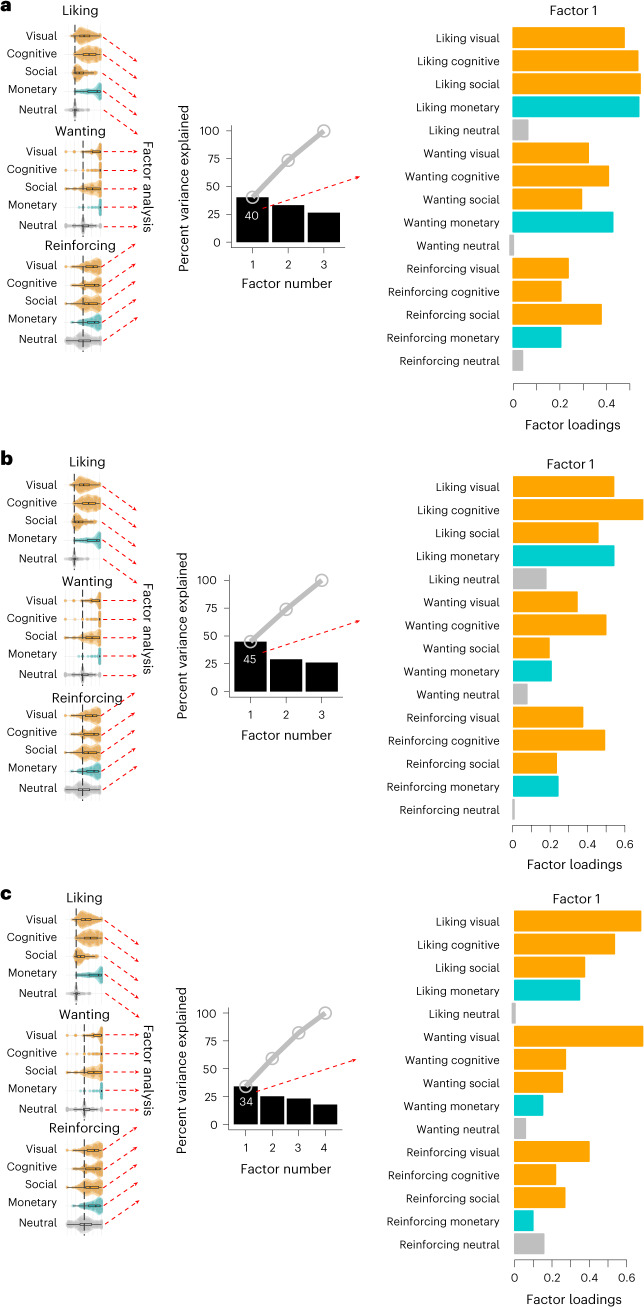
Intrinsic reward sensitivity is partially domain general. **a**–**c**, The variance explained by the first factor of a factor analysis (using a bi-factor rotation) across all reward types (visual, cognitive, social, monetary, neutral) and responses (liking, wanting, reinforcing) shows a clear first factor that accounts for, on average, about 40% of the variance in responses in experiments 1 (**a**, *N* = 132 participants), 2 (**b**, *N* = 171 participants) and 3 (**c**, *N* = 180 participants). The corresponding loadings are displayed with orange bars for intrinsic rewards, blue bars for monetary reward and grey bars for neutral stimuli. We term this first factor reward sensitivity. Note that the violin plots on the left correspond to Fig. 2 and are used to illustrate the data used in the factor analysis (see Fig. 2 for details).

To address this question, we conducted a factor analysis with a bi-factor rotation (see Methods) across all measures (liking, wanting and reinforcement) and all stimuli (visual, cognitive, social, monetary and neutral), after having checked data factorability using the overall Measure of Sampling Adequacy (MSA) (which were all above 0.5; experiment 1, MSA = 0.63; experiment 2, MSA = 0.64; experiment 3, MSA = 0.63; see Supplementary Fig. 2). We were interested in whether a latent variable related to all of the rewards but the neutral stimuli could be observed, which partially explains the individual differences. The number of factors was determined with parallel analyses (see Methods). The factor analysis revealed a first factor (among 3 in experiments 1 and 2, and among 4 in experiment 3) that included high positive loadings on all the rewards but not on the neutral stimuli. We refer to the first factor as reward sensitivity, which accounted for about 40% of individual differences in responses to rewards (experiment 1, 40%; experiment 2, 45%; experiment 3, 34%; Fig. 3a–c and Supplementary Fig. 3). This suggests that responses are partially domain general across reward categories. Note that the intrinsic reward loadings tended to be higher than monetary reward loadings in the first factor (especially in experiments 2 and 3), which suggests that the first factor is more dominated by intrinsic rewards and that other factors may capture the remaining sensitivity to monetary rewards (see Supplementary Fig. 3 for additional factors).

### Reward sensitivity is related to mental health

So far we find that responses to intrinsic rewards (liking, wanting and reinforcing) are domain general. We next asked whether the reward sensitivity score is related to affective aspects of mental health. That is, do people with high reward sensitivity experience better affective health?

**Fig. 4:**
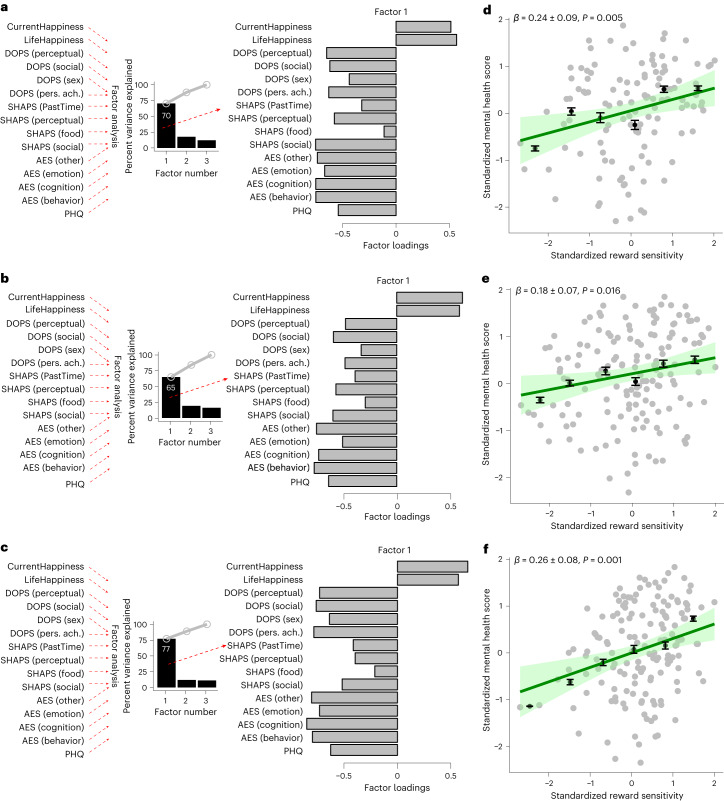
Mental health score is associated with reward sensitivity. **a**–**f**, A factor analysis was performed across all questionnaire sub-scales (PHQ, patient health questionnaire; AES, apathy evaluation scale; SHAPS, Snaith–Hamilton pleasure scale; DOPS, domain of pleasure scale) as well as life satisfaction and happiness, to reduce the dimension of the scores and ratings to a single factor score. The first factor explained about 70% of the variance in experiments 1 (**a**, *N* = 132 participants), 2 (**b**, *N* = 171 participants) and 3 (**c**, *N* = 180 participants) with similar loadings across studies. Negative loadings correspond to questionnaire scores that are negatively correlated with the factor; for example, as expected, a high depression score (PHQ) loads negatively on the mental health score. Positive loadings correspond to a positive correlation with mental health scores; for example, as expected, the happiness rating has a positive weight on the mental health score. Reward sensitivity score predicted mental health score, correcting for demographics (age, gender, qualifications, income, marital status) and IQ for experiments 1 (**d**), 2 (**e**) and 3 (**f**). Grey dots represent participants. Black dots represent the median of binned scores (for illustration purposes only). Error bars = s.e.m. Model prediction (of all data points) is represented by green line, with shaded areas corresponding to the 95% confidence bounds. *β* ± s.e.m. represent the linear regression (also including demographics) coefficients with the corresponding standard error and are tested against 0 using a two-sided *t*-test. Pers. ach., personal achievement.

To assess affective health, we used rating of current happiness, rating of life satisfaction, as well as all sub-scale scores (if such exist, otherwise the full score), including the apathy evaluation scale (AES), Snaith–Hamilton pleasure scale (SHAPS), domain of pleasure scale (DOPS) and patient health questionnaire (PHQ). We performed the same factor analysis with a bi-factor rotation on all of these measures to extract a latent variable that would be related to mental health. The factor analysis revealed a first factor (among 3) that explained 70% of the variance in mental health score in experiment 1, 65% in experiment 2 and 77% in experiment 3. Loadings were positive for happiness and life satisfaction measures and negative for the rest (Fig. 4a–c). The score for this factor is therefore indicative of affective aspects of mental health (for brevity, we will refer to this score as the mental health score going forward).

We estimated a linear model with mental health score as the dependent variable and reward sensitivity as the independent variable, controlling for age, gender, education, income and IQ. This analysis revealed that reward sensitivity was associated with mental health score controlling for demographics and IQ (experiment 1, *β* = 0.24 ± 0.09, *t*(112) = 2.8, *P* = 0.005; experiment 2, *β* = 0.18 ± 0.07, *t*(149) = 2.4, *P* = 0.016; experiment 3, *β* = 0.26 ± 0.08, *t*(145) = 3.4, *P* = 0.001). Not controlling for any factor shows comparable results (experiment 1, *β* = 0.24 ± 0.09, *t*(118) = 2.7, *P* = 0.008; experiment 2, *β* = 0.18 ± 0.08, *t*(155) = 2.2, *P* = 0.027; experiment 3, *β* = 0.26 ± 0.08, *t*(152) = 3.4, *P* = 0.001; Fig. 4d–f).

To assess whether the relationship between mental health score and reward sensitivity was present for both intrinsic and monetary rewards separately, we performed two-factor analyses on the behavioural responses: one excluding monetary rewards and the other excluding all intrinsic rewards (visual, cognitive and social; see Supplementary Fig. 4). We then related these new reward sensitivity scores to mental health score. Across all experiments we found that intrinsic reward sensitivity predicted mental health score controlling for demographics or not (see Table 1), whereas monetary reward sensitivity did not (see Table 1). The models using intrinsic reward sensitivity as the predictor predicted mental health scores better than the corresponding models with material reward sensitivity, as observed by lower Bayesian Information Criterion (BIC) scores for the former (Table 1). These results suggest that sensitivity to intrinsic rewards alone may be more important to predict mental health score than monetary reward sensitivity, which does not help to predict mental health score.

**Table 1:** Intrinsic reward sensitivity, but not monetary reward sensitivity, is associated with mental health score

Experiment 1						
Statistics	*β*	s.e.m.	df	*t*-value	*P*-value	BIC
Regression						
Intrinsic reward (dem.)	0.21*	0.09	113	2.79	0.020	295.21
Monetary reward (dem.)	0.13	0.11	113	2.19	0.235	299.51
Intrinsic reward (no dem.)	0.19*	0.08	119	2.87	0.025	282.93
Monetary reward (no dem.)	0.11	0.11	119	2.26	0.293	286.96

Experiment 2						
Intrinsic reward (dem.)	0.23*	0.07	147	3.59	0.0010	351.96
Monetary reward (dem.)	0.04	0.10	147	2.55	0.7110	363.11
Intrinsic reward (no dem.)	0.20*	0.07	153	3.42	0.0048	361.07
Monetary reward (no dem.)	0.05	0.10	153	2.40	0.6105	368.90

Experiment 3						
Intrinsic reward (dem.)	0.18*	0.09	155	2.84	0.039	419.14
Monetary reward (dem.)	−0.11	0.07	155	3.26	0.144	421.38
Intrinsic reward (no dem.)	0.18*	0.09	162	2.82	0.040	411.20
Monetary reward (no dem.)	−0.10	0.07	162	3.32	0.173	413.62

Intrinsic reward sensitivity is associated with mental health score in experiments 1 (*N* = 132 participants)*,* 2 (*N* = 171 participants) and 3 (*N* = 180 participants), whereas monetary reward sensitivity is not. dem., controlling for demographics and IQ; no dem., not controlling for demographics and IQ; *β*, beta coefficient; s.e.m., standard error of the mean; df, degrees of freedom; BIC is a measure of model fit penalizing complexity, with lower BICs representing better model evidence. The *P*-value refers to a two-sided *t*-test.

^*^P< 0.05.

Intrinsic reward sensitivity as used in the previous analysis was computed using three reward types (visual, cognitive, social), whereas monetary reward sensitivity was computed using only one. To ensure that the finding is not contingent on this difference, we applied the same factor analyses to all the measures (liking, wanting and reinforcement) and all the reward pairs (visual–monetary, cognitive–monetary, social–monetary, visual–cognitive, visual–social, social–cognitive) plus the neutral stimuli (to make sure the general factors still are reward-specific factors). The resulting first factor corresponds to a reward sensitivity to that given pair. A linear model with this reward sensitivity was then fit to the mental health score. In total, we estimated six linear models to fit mental health score to compare two balanced model families: three models including monetary rewards and three models only including intrinsic rewards. The BIC scores were summed across models within a family and then compared. Results revealed that the family that included intrinsic rewards had lower BIC than the model family that included monetary reward (see Table 2).

**Table 2:** Mental health is associated more closely with intrinsic reward sensitivity than with monetary reward sensitivity

	Experiment 1		Experiment 2		Experiment 3	
	Controlled (demographics and IQ)	Not controlled	Controlled (demographics and IQ)	Not controlled	Controlled (demographics and IQ)	Not controlled
	BIC	BIC	BIC	BIC	BIC	BIC
**Intrinsic reward**	1,126	1,149	1,485.7	1,456.4	1,541.3	1,525.4
**Monetary reward**	1,133	1,159	1,487.6	1,457.8	1,546.8	1,531.2

In experiments 1 (*N* = 132 participants), 2 (*N* = 171 participants) and 3 (*N* = 180 participants), we compared how well mental health scores were explained by a family of three models that extracted reward sensitivity from pairs of intrinsic rewards to a family of three models that extracted reward sensitivity from pairs of monetary and intrinsic rewards. The former had lower BIC scores which means better model evidence. All models also included the same neutral stimuli.

Overall, the results suggest that sensitivity to intrinsic rewards is partly domain general. The common sensitivity across intrinsic rewards, but not monetary rewards, is associated with affective aspects of mental health.

## Discussion

Intrinsically rewarding activities include a large variety of human behaviours, ranging from exploring nature and consuming art to reading books. Here we show that the sensitivity to diverse visual, cognitive and social, putative intrinsically rewarding stimuli is domain general and related to mental health. All of these rewards (but not neutral stimuli) triggered the same type of responses as the secondary (monetary) reward. They elicited positive feelings (were ‘liked’), generated approach/consummatory behaviours (were ‘wanted’) and increased the likelihood of the action that led to them (were ‘reinforcing’). These three responses have been suggested to be characteristic of a reward^13,19^. A factor analysis performed on these responses across all stimuli revealed that ~40% of the variance in behaviour was explained by a first factor corresponding to sensitivity to all rewards, but not to the neutral stimulus. This suggests that sensitivity to intrinsic (and non-intrinsic) rewards is partly domain general. Intriguingly, individual differences in reward sensitivity was associated with affective aspects of mental health. This association was largely driven by intrinsic rewards sensitivity, rather than by monetary reward sensitivity. In fact, while sensitivity to intrinsic rewards was associated with mental health, sensitivity to monetary rewards was not.

Vast research on decision-making in humans and non-human animals has focused on primary (for example, water, food) and secondary (for example, money) rewards, with studies reporting mixed results regarding the link between features of mental health and sensitivity to monetary rewards^3,14^^,^^20–64^. Our findings suggest intrinsic reward processing may be more vital for well-being and thus highlight the importance of studying rewards beyond primary and secondary. Crucially, our finding may help explain thriving and suffering; individuals with high sensitivity to the common rewarding features of different intrinsically rewarding activities will be more inclined to engage in a variety of seemingly diverse activities than those with low sensitivity. Over the long term, the former may experience better mood and find activities that they excel at, whereas others will be less active, performing mostly essential tasks^4,65^. Indeed, the concept of thriving, which describes a person who is fully functioning in mental, physical and social terms^66^, includes positive affective health.

An open question is what are the common features that are rewarding across the diverse stimuli studied here? One possibility is that intrinsic rewards generate feelings of self-efficacy, that is, of autonomy and competence^67,68^. Indeed, this seems to be the case of many intrinsically rewarding activities, such as solving crossword puzzles, consuming arts, playing sports and helping others^2^. Even intrinsic rewards that, on the surface, do not seem to offer much in the way of increased self-efficacy, may do exactly that on closer inspection. Staring at landscapes, for example, allows a relaxed state for consolidating thoughts^69^ and learning that others agree with us may give a sense of competence. This in turn may increases mental health^70^, or the relationship may be reversed (mental health may lead to greater intrinsic reward sensitivity), or alternatively mediated by a third factor. Although we focused here on affective aspects of mental health, other aspects of mental health may also be associated with intrinsic reward sensitivity.

Another common feature of the intrinsic rewards could be that they trigger curiosity^71^, that is, one may desire to consume information (corresponding to epistemic uncertainty reduction) more than string letters, and view landscapes (corresponding to perceptual uncertainty reduction) more than walls (for example, see ref. ^72^) because of curiosity. Although it is less clear why people would be more curious about others who agree with them than others than disagree with them, people who are more responsive to intrinsically rewarding stimuli may be more curious. Curiosity in turn is associated with mental health^73^.

Our finding that sensitivity to intrinsic rewards is partly domain general triggers the hypothesis that they share common neural fingerprints. Indeed, common neural responses to different types of material rewards^74–77^, primary rewards and intrinsic rewards^9,78,79^ have been observed in the ventro-medial pre-frontal cortex and the ventral striatum. This suggests that intrinsic reward sensitivity may partly rely on the same neural system as material and primary rewards. It is also likely, however, that the brain has a system in place to distinguish between intrinsic and material rewards^80^, as well as between the value of different intrinsic rewards. The latter may account for the non-shared sensitivity (~60%) to different rewards and different preferences across individuals for engaging in different rewarding activities.

A limitation of experiments 1 and 2 is that our measures (‘liking’, ‘wanting’, ‘reinforcing’ strength) were presented in a fixed order that may lead to one response systematically impacting the other. To address this, we conducted experiment 3, in which measurements were presented in random order. Another limitation of experiments 1 and 2 is that both liking and wanting were measured using a rating scale, which may have contributed to the measures being related. We addressed this in experiment 3, in which liking was measured using a rating scale and wanting was measured using a choice task. Experiment 3 replicated the results of experiments 1 and 2, strengthening the conclusions. Furthermore, a limitation of all studies is that we test for a correlation, not a causation. We therefore cannot conclude whether sensitivity to intrinsic rewards alters mental health, or vice versa, or if a common third variable modulates both. Finally, future studies may test different types of intrinsic rewards (such as music and sports) and different types of primary rewards (such as food) to ascertain generalizability.

Although a large body of literature has been dedicated to the empirical study of primary and secondary rewards, the empirical study of intrinsic rewards is still in its infancy (for recent work see refs. ^7,11,81–85^). This line of research is challenging as intrinsic rewards are difficult to quantify, yet critical for a full and deep understanding of the human experience.

## Methods

For all experiments, ethical approval was provided by the Research Ethics Committee at University College London (project no. 3990/003) and all participants gave written informed consent to participate.

### Statistics and reproducibility

#### Participants (experiment 1)

We estimated an effect size of about 0.25 on the basis of a pilot study. We therefore needed a sample size of 128 for a regression with a power of 80% and significance level *α* = 0.05. We added around 15% to account for failed attention checks, which resulted in 149 participants. Data were collected between 22 and 29 November 2021.

One hundred and fourty-nine participants completed the task on Prolific (https://www.prolific.co/) online system (see the flow chart in Supplementary Fig. 1); 17 participants failed the comprehension and/or attention checks, and thus their data were not analysed (see details below). Data of 132 participants were thus analysed (female = 34%, age = 33 ± 12 (M ± s.d.); male = 66%, age = 29 ± 9; other = 0%). Ethnicity data in all experiments was not collected as it was not planned. Participants received £7.50 per hour for their participation plus a 50 pence bonus payment. The experiment lasted around 90 min.

#### Participants (experiment 2)

The sample size was based on a power analysis based on experiment 1, which showed that 159 participants were required for a regression weight of 0.22 (the lower bound of the effect size from the regression between mental health score and reward sensitivity, without correcting for demographics, which was 0.23 ± 0.01 in experiment 1) with 80% power and *α* = 0.05. We anticipated that about 30 participants would fail the attention and/or comprehension checks. We therefore recruited 188 participants on the Prolific website (see the flow chart Supplementary Fig. 1). Data of 17 participants were not analysed as they did not pass the comprehension and/or attention checks. Thus, 171 participant’s data was analysed (female = 51%, age = 34 ± 16 (M ± s.d.); male = 49%, age = 35 ± 13; other = 0%). Participants received £7.50 per hour for their participation. Data were collected between 20 and 25 January 2022.

#### Participants (experiment 3)

The sample size was calculated as per experiment 2. As we added more catch trials (in the wanting measure, see below), we expected a higher rate of failure for the attention checks. We therefore added approximately 40 additional participants and recruited 198 participants on Prolific website (see the flow chart in Supplementary Fig. 1). Data of 18 participants were not analysed as they did not pass comprehension and/or attention checks; thus, 180 participants’ data were analysed (female = 41%, age = 38 ± 12 (M ± s.d.); male = 59%, age = 39 ± 12; other = 0%). Participants received £7.50 per hour for their participation. Data were collected between 18 and 31 January 2023.

#### Comprehension/attention checks

The following checks were employed:

(i) On six trials throughout the experiment, participants were asked to move the rating scale towards one of the sides (for example, ‘Please move the cursor to the left side’);
(ii) Following the initial instructions of each reward type in experiments 1 and 2, participants were asked which letter corresponded to which reward (for example, ‘What is associated with “L”: landscape or wall?’).
(iii) During each questionnaire, participants were asked to select a particular answer on one trial (for example, ‘Please select answer two’).
(iv) For experiment 3, participants were asked four times whether they already have seen the stimulus currently displayed on the screen during the wanting session (half were repeated and half were new).

The data of participants who failed more than two trials were not analysed.

### Stimuli (experiments 1 and 2)

We selected stimuli thought to be intrinsically rewarding from three different domains (visual, cognitive, social). This was done to assess domain-general reward sensitivity. Each reward type was paired with a stimulus from the same domain thought not to be rewarding. Furthermore, a secondary reward was used: money, with no money as an alternative. Finally, a non-rewarding stimuli pair was also included as an additional control. All of these are described in detail below.

The visual rewards were pictures of landscapes extracted from the OASIS image set (https://osf.io/6pnd7/). Landscapes are believed to be rewarding^7^ and are associated with positive emotions^17^. They were contrasted with pictures of walls, which are not thought to be rewarding and are emotionally neutral^17^. These were associated with the letter L (for landscape) and W (for wall).

The cognitive rewards were informative sentences extracted from the *Encyclopedia Britannica Trivia* (https://www.britannica.com/). It has been shown^86–88^ that humans select to observe (and thus read) sentences, and that the opportunity to consume such knowledge activates the reward system^86–88^.The corresponding alternative stimuli were the same sentences, except that the order of the letters was scrambled, resulting in a non-informative string of letters. These were associated with the letter I (for information) and O (for string of letters).

The social reward was a confirmation of participants’ preferences that were measured at the beginning of the experiment (see ‘Procedure’ section below). That is, another player (represented by the neutral name Addison) shared 4/5 of the participant’s preferences. The control stimulus was a player (represented by the neutral name Daryl) who shared 1/5 of the participants’ preference. The name Addison or Daryl was displayed on the screen for 1,500 ms before a pair of items was displayed (for example, the words ‘coffee’ and ‘tea’). The other player’s preference was indicated by a rectangle surrounding the preferred item (for example, coffee). Studies have shown the existence of a confirmation bias by which subjects select to observe information that they suspect confirms their beliefs (for example, ^18,89^), and confirmation has been shown to activate the reward system^11^.

Participants were told that the monetary reward was 1.5 pence, which was to be added to their bonus payment. This was represented by a picture of a £1 coin. In reality, though, all participants received the same bonus of 50 pence at the end of the study. The alternative stimulus was not receiving a bonus, represented by a red crossed on top the £1 coin. These were associated with the letter R (for reward) and Z (for zero).

Finally, we used vertical and horizontal black lines on a white background as non-rewarding neutral stimuli. The number of vertical or horizontal lines per picture varied between 1 and 126. These were associated with the letter V (for vertical) and H (for horizontal).

The use of letters was intended to control for the word length across rewards and alternatives.

### Stimuli (experiment 3)

In experiment 3 the same stimuli were used as in experiments 1 and 2 except:

(i) Words (for example, LANDSCAPE) were used instead of letters (for example, L).
(ii) Coin pictures varied from trial to trial. This was done to ensure the results in experiment 1 and 2 were not due to the coin stimulus being always exactly the same.

### Procedure

Participants first indicated their preferred item among each of 44 different unique pairs of items (for example, ‘coffee’ or ‘tea’) using the left and right keys, with no repeated item. This was self-paced. These preferences subsequently informed the social rewards that would appear later (see below).

Participant then completed three blocks in the following order:

1. **Liking**. Liking refers to the hedonic response to reward consumption. The purpose of this rating was to measure how much participants liked a rewarding stimulus relative to a domain-similar alternative. There were 20 blocks comprising two blocks of each of the five intrinsic rewards and two blocks of each of the five alternatives (2 × 5 + 2 × 5 = 20). Intrinsic rewards and their alternatives followed each other (either: intrinsic–alternative–intrinsic–alternative or alternative–intrinsic–alternative–intrinsic). Each block included five trials in which the subject experienced the stimulus, and a liking scale was introduced thereafter. The order of domains was randomized. Each block consisted of a letter that was displayed for 1,500 ms before the corresponding stimulus. This pairing appeared five times, after which the participant indicated how much they liked the block using a continuous scale with six labels from 0 (disliked a lot) to +100 (liked a lot), self-paced. The duration of the stimuli on screen was contingent on processing time (that is, reading a sentence takes longer than perceiving a picture) and was as follows: 5,000 ms for a cognitive reward (information or random strings of letter), 2,000 ms for a social reward (agreement or disagreement), 2,000 ms for a visual reward (landscape or wall), 2,000 ms for a non-reward neutral stimuli (vertical or horizontal), and 1500 ms for the monetary reward (coin or no coin). We quantified liking for a given reward (for example, a landscape versus a wall) as the contrast between the liking rating after the a priori rewarding stimulus blocks, and the liking rating after the a priori defined not rewarding stimuli blocks.
2. **Wanting**, **experiments 1 and 2**. Wanting refers to the appetitive response to a reward, that is the willingness to approach it. The purpose of this task was to measure how much participants wanted to engage with a rewarding stimulus relative to a domain-similar alternative. Participants indicated how much they wanted to see ten trials of the reward stimulus (represented by its letter, for example, L) or ten trials of the alternative stimulus (for example, W) using a continuous scale with seven labels from definitely ten trials of alternative stimuli to definitely ten trials of reward. This was self-paced. They were then exposed to ten trials of the chosen stimulus (and ten trials of alternative if they did not move the cursor). This rating directly corresponded to the contrast between the a priori rewarding stimuli and the a priori not rewarding stimuli and was therefore used as such to quantify wanting.
  **Wanting**, **experiment 3**. On each of 15 trials, participants chose between observing the reward stimulus (in words, for example, LANDSCAPE) or the alternative (for example, WALL) using the left or right key. This was self-paced. They were then exposed to the chosen stimulus. We used this choice response to test for the generalizability of the results of experiments 1 and 2, which used a Likert scale. The proportion of chosen a priori rewarding stimuli (versus a priori not rewarding stimuli) was used to quantify wanting.
3. **Reinforcement Learning**. The purpose of this task was to measure the reinforcing strength of intrinsic rewards, that is the extent to which a cue leading more often to an intrinsic reward is chosen. Participants performed one block of a probabilistic instrumental learning task with 24 trials for each stimulus domain (the learning phase). On each trial two abstract cues appeared side by side, each was probabilistically linked to a reward stimulus (that is, 0.75/0.25) or a domain-similar alternative (that is 0.25/0.75). Participants then selected between the abstract cues (self-paced) after which their choice was confirmed for 800 ms and then the associated letter appeared for 1,500 ms, followed by the stimulus for 1,500 ms for monetary reward, 2,000 ms for the visual and social rewards and the neutral stimuli, 5,000 ms for the cognitive reward. The proportion of chosen cue leading to the a priori rewarding stimuli (versus to the a priori not rewarding stimuli) was used to quantify reinforcement.

Finally, participants completed self-report questionnaires at the end of the experiment which assess different aspects of affective health including a large range of depression symptoms, mood, motivation, anhedonia, anticipated and experienced pleasure, life satisfaction and current happiness. These included: the nine-item version Patient Health Questionnaire (PHQ-9^90^), which covers the DSM-IV criteria for major depressive disorder; AES^91^, which is commonly used to measure apathy^92^; SHAPS^93^, to measure anhedonia^94^; and DOPS, which measures experienced pleasure (as opposed to motivational and anticipatory pleasure)^95–97^. Participants also answered two happiness questions^98^: ‘Overall, how satisfied are you with your life nowadays?’; and^3,99^ ‘How happy are you right now?’ at the beginning of the experiment.

### Statistics

We were interested in assessing whether participants liked each reward type (referred to as liking), whether they wanted them (referred to as wanting) and whether they were reinforcing (referred to as reinforcing)^13^.

Liking was quantified by subtracting the average liking rating after the alternative blocks from the liking rating after the reward blocks. The wanting measure was equal to the cursor position, which could vary from 0 (the participant definitely wanted to be exposed to ten trials of control stimuli) to 100 (the participant definitely wanted to be exposed to ten trials of rewarding stimuli). The reinforcing strength of each reward was assessed by the percentage of times the participants chose the cue leading more often to the rewarding stimulus. We used non-parametric (two-sided Wilcoxon signed rank tests) to test whether the variables are different from the null hypothesis (that is, reward versus control for liking, 50% for wanting, and the reinforcing strength). The data met the assumptions for this test.

A reward sensitivity factor was extracted for all responses (liking, wanting and reinforcing) across the five stimulus types using an exploratory factor analysis performed using the R ‘fa’ function from the psych library. This factor analysis used the maximal likelihood to find best solution. We used a bi-factor rotation criterion designed to produce a rotated loading matrix that has an approximate bi-factor structure, a general factor and a number of group factors^100^. Each factor is a linear combination of the original variables. The resulting loadings are interpreted as the coefficients of the linear combination of the initial variables from which the factors are constructed. Positive loadings indicate that a variable and a factor are positively correlated, whereas negative loadings indicate a negative correlation; null loadings mean no correlation between a variable and a factor^101^. For example, a factor with high positive loading for all rewards but the neutral stimuli means that all the rewards are strongly positively correlated with that factor. The explained variance for such a factor was used as a measure of domain generality. The resulting reward sensitivity score was the representation of the data in the factor space for the first (and general) factor.

Similarly, a mental health score, reflecting affective factors of mental health, was extracted from the questionnaire scores (AES^91^, SHAPS^93^, DOPS^95^ and PHQ^90^) using a similar factor analysis. Questionnaire subscales were used together with score of life satisfaction and current happiness.

To assess whether general reward sensitivity is associated with mental health score, we run a regression including standardised general reward sensitivity as an independent variable, and IQ and demographics (age, gender, income, and marital status) as covariates, and the standardized mental health score as a dependent variable. Linear models were estimated using the R (v.4.2.2) ‘lm’ function. Influential points is the regressions were removed using the difference in fit(s) (DFFITS) method^102^. All of the reported tests are two-sided. The data met the assumptions of the regressions (that is linearity and homoscedasticity).

We used standard model comparison techniques^103,104^ to compare linear model fits. For each model fit, we computed BIC, which penalizes for model complexity (that is number of parameters). The model with the lowest BIC is the preferred model (as long as the BIC difference is larger than two, which was always the case in our results). For the family model comparison, we summed BIC across models belonging to the same family (with an equal number of models per family, which ensure a fair comparison).

The following R libraries were used to analyse the data and to plot the figures: pwr, dplyr, tidyr, ggplot2, patchwork, nFactors, psych, corrplot, car and lmtest.

### Reporting summary

Further information on research design is available in the Nature Portfolio Reporting Summary linked to this article.

### Supplementary information

Supplementary Information Supplementary Figs. 1–4 and Tables 1–3.

Reporting Summary

## Data Availability

Data and analysis code are available on Github (https://github.com/BastienBlain/SensitivityToIntrinsicRewardsIsDomainGeneralAndRelatedToMentalHealth-).

## Code availability

The analysis code is available on Github (https://github.com/BastienBlain/SensitivityToIntrinsicRewardsIsDomainGeneralAndRelatedToMentalHealth-). Visual stimuli (landscapes and walls) are from OASIS (https://osf.io/6pnd7/).
