# Supplementary material for: Sensitivity to intrinsic rewards is domain general and related to mental health
Source: Nat Ment Health. 2023 Sep 6;1(9):679–91. doi: 10.1038/s44220-023-00116-x (PMC11041740; doi:10.1038/s44220-023-00116-x)
Supplement: Supplementary file 1 — Supplementary Figs. 1–4 and Tables 1–3. [file 44220_2023_116_MOESM1_ESM.pdf]

nature mental health

Article

https://doi.org/10.1038/s44220-023-00116-x

Sensitivity to intrinsic rewards is domain general and related to mental health

In the format provided by the authors and unedited

Supplementary Material

All putative rewards are more liked, wanted and reinforcing than their alternatives

A Experiment 1

Liking

Stimulus type	Reward (M)	Reward (SE)	Alternative (M)	Alternative (SE)	Delta (M)	Delta (SE)	r-value	p-value
Visual	83.00	1.48	42.10	1.86	40.90	2.38	1.06	8E-34
Cognitive	79.39	1.38	22.95	1.63	56.43	2.14	1.19	1E-42
Social	70.48	1.50	47.85	1.61	22.64	1.20	0.80	4E-20
Monetary	88.71	1.43	15.34	1.62	73.37	2.16	1.21	5E-44
Neutral	55.45	1.76	52.64	1.86	2.81	2.56	0.10	0.26

Wanting

Stimulus type	Reward (M)	Reward (SE)	r-value	p-value
Visual	87.62	1.81	0.82	3.1E-21
Cognitive	93.42	1.46	0.88	7.65E-24
Social	75.56	2.16	0.70	8.46E-16
Monetary	97.30	0.88	0.95	1.05E-27
Neutral	50.77	2.30	0.02	0.83

Reinforcement

Stimulus type	Reward (M)	Reward (SE)	r-value	p-value
Visual	73.99	1.94	0.72	9.52E-17
Cognitive	76.61	1.86	0.76	1.88E-18
Social	68.84	2.00	0.63	3.92E-13
Monetary	77.71	1.72	0.80	5.23E-20
Neutral	51.86	2.41	0.06	0.47

B Experiment 2

Liking

Stimulus type	Reward (M)	Reward (SE)	Alternative (M)	Alternative (SE)	Delta (M)	Delta (SE)	r-value	p-value
Visual	84.19	1.24	40.32	1.61	43.86	2.04	1.25	5.35E-47
Cognitive	76.05	1.49	20.99	1.52	55.06	2.12	1.29	2.29E-49
Social	68.08	1.38	46.70	1.46	21.38	2.01	0.87	1.75E-23
Monetary	87.42	1.32	16.85	1.49	70.58	1.99	1.37	1.21E-55
Neutral	50.85	1.72	50.46	1.76	0.38	2.46	0.046	0.59

Wanting

Stimulus type	Reward (M)	Reward (SE)	r-value	p-value
Visual	90.22	1.50	0.85	7.05E-29
Cognitive	92.96	1.39	0.90	1.13E-31
Social	75.16	1.85	0.69	1.68E-19
Monetary	96.33	0.87	0.94	1.59E-34
Neutral	50.78	1.98	0.03	0.66

Reinforcement

Stimulus type	Reward (M)	Reward (SE)	r-value	p-value
Visual	72.44	1.71	0.70	9.90E-20
Cognitive	74.83	1.76	0.72	3.59E-21
Social	65.79	1.82	0.55	8.42E-13
Monetary	76.92	1.54	0.78	2.18E-24
Neutral	51.36	2.04	0.05	0.56

C Experiment 3

Liking

Stimulus type	Reward (M)	Reward (SE)	Alternative (M)	Alternative (SE)	Delta (M)	Delta (SE)	r-value	p-value
Visual	83.80	1.14	39.28	1.44	44.52	1.84	1.31	7.59E-51
Cognitive	78.12	1.26	16.31	1.31	61.81	1.82	1.39	1.72E-57
Social	74.55	1.13	39.81	1.29	34.74	1.72	1.24	5.05E-46
Monetary	71.38	1.47	17.22	1.53	54.16	2.12	1.31	2.27E-51
Neutral	47.13	1.60	45.13	1.68	1.99	2.32	0.10	0.25

Wanting

Stimulus type	Reward (M)	Reward (SE)	r-value	p-value
Visual	83.56	1.95	0.78	2.38E-25
Cognitive	94.41	1.03	0.90	1.51E-33
Social	80.63	1.81	0.77	8.24E-25
Monetary	92.04	1.10	0.87	2.54E-33
Neutral	52.52	2.60	0.76	0.31

Reinforcement

Stimulus type	Reward (M)	Reward (SE)	r-value	p-value
Visual	73.59	1.84	0.67	2.53E-19
Cognitive	81.41	1.42	0.80	4.03E-27
Social	73.08	1.96	0.63	1.64E-17
Monetary	80.19	1.39	0.80	6.73E-27
Neutral	49.40	2.39	0.02	0.75

Table S2. All putative rewards are more liked, wanted and reinforcing than their alternatives in Experiment 1 (A, N = 132 participants), 2 (B, N = 171 participants), & 3 (C, N = 180 participants). *M = mean, SE = standard error of the mean. Liking corresponds to liking ratings from 0 to 100, wanting corresponds to wanting rating (ratings higher than 50 means that participants wanted to be exposed to a 10-trials block of rewards for Experiment 1 & 2) or the proportion of trials in which participants chose to be exposed to the reward versus the alternative stimulus (Experiment 2) Reinforcement corresponds to percentage of times subjects selected the cue which leads more often to the reward. df = degree of freedom. Delta corresponds to the difference between the liking rating for the reward and the liking rating for the alternative stimulus. R values corresponds to Mann-Whitney-U-test effect size and p values are statistics from a two-sided Wilcoxon signed-rank test comparing delta to zero for liking rating, and to 50 otherwise.*

Liking comparison across all stimuli

Experiment 1

Reward \ Control	Wall	Letters	Disconfirmation	No coin	Horizontal
Landscape	40.9±2.3	60±2.3	35.2±2.1	67.7±2.3	30.4±2.2
Information	37.3±2.3	56.4±2.5	31.5±2.2	64±2.2	26.7±2.1
Confirmation	28.4±2.3	47.5±2.3	22.6±2	55.1±2.5	17.8±2
Coin	46.6±2.3	65.8±2.4	40.9±2.1	73.4±2.7	36.1±2.2
Vertical	13.4±2.3	32.5±2.2	7.6±2.3	40.1±2.4	2.8±1.2

Experiment 2

Reward \ Control	Wall	Letters	Disconfirmation	No coin	Horizontal
Landscape	43.9±2.1	63.2±2.2	37.5±1.9	67.3±2.1	33.7±2
Information	35.7±2.2	55.1±2.2	29.3±1.9	59.2±2.1	25.6±2.3
Confirmation	27.8±2.1	47.1±1.9	21.4±1.6	51.2±2	17.6±2.1
Coin	47.1±2.2	66.4±2.2	40.7±2	70.6±2.4	37±2.2
Vertical	10.5±2	29.9±2.1	4.1±2.1	34±2.2	0.4±1.1

Experiment 3

Reward \ Control	Wall	Letters	Disconfirmation	No coin	Horizontal
Landscape	44.5±2	67.5±2	44±1.9	66.6±2.2	38.7±2
Information	38.8±2	61.8±2.1	38.3±2	60.9±2.2	33±2
Confirmation	35.3±2	58.2±2	34.7±1.9	57.3±2.1	29.4±1.9
Coin	32.1±2.1	55.1±2.1	31.6±2.1	54.2±2.3	26.2±2.2
Vertical	7.8±2	30.8±1.9	7.3±2	29.9±2.1	2±1.6

Table S2*. *All rewards (row) were significantly more liked than all* alternative *(column) in Experiment 1 (N = 132 participants), 2 (N = 171 participants), & 3 (N = 180 participants). *Values represent the mean liking difference between a given rewarding stimulus and a given* alternative, *and the standard error of that difference (M±SE). For example, the value in the second row and third column means that information is liked more than disconfirmation which is the* alternative *for the social reward. All values are compared using two-sided Wilcoxon signed rank tests and were*

significantly different from 0 at p < 10^{-14}, except Vertical versus Horizontal (Experiment 1 (p = 0.11), Experiment 2 (p = 0.4), Experiment 3 (p = 0.09)) and Vertical versus wall and disconfirmation (Experiment 1, wall: p = 1.64E-7, Disconfirmation: p = 0.006; Experiment 2, wall: p = 1.32E-7, Disconfirmation: p = 0.054;, Experiment 3, wall: p = 1.44E-5, Disconfirmation: p = 0.0007).

Neutral stimuli (vertical and horizontal lines) are less liked than any other reward

A Experiment 1

Δ Liking (reward – alternative) – (vertical – horizontal)

Stimulus type	Reward (M)	Reward (SE)	r-value	p-value
Visual	38.09	2.57	0.82	6.26E-21
Cognitive	53.63	2.77	0.85	2.12E-22
Social	19.83	2.24	0.69	1.47E-15
Monetary	70.56	2.91	0.87	2.79E-23

B Experiment 2

Δ Liking (reward – alternative) – (vertical – horizontal)

Stimulus type	Reward (M)	Reward (SE)	r-value	p-value
Visual	43.48	2.36	0.84	4.23E-28
Cognitive	54.68	2.37	0.86	2.74E-29
Social	21.00	1.76	0.75	1.43E-22
Monetary	70.19	2.56	0.86	2.66E-29

C Experiment 3

Δ Liking (reward – alternative) – (vertical – horizontal)

Stimulus type	Reward (M)	Reward (SE)	r-value	p-value
Visual	42.53	2.58	0.83	1.20E-28
Cognitive	59.82	2.63	0.85	2.10E-30
Social	32.75	2.51	0.78	7.29E-26
Monetary	52.17	2.84	0.84	2.98E-29

Table S3. Neutral stimuli (vertical versus horizontal lines) are less liked than any other reward (reward versus alternative) in Experiment 1 (A, N = 132 participants), 2 (B, N = 171 participants), & 3 (C, N = 180 participants)*. M = mean, SE = standard error of the mean. Values below the reward (M) column correspond to the difference of differences. That is, the liking rating between the reward in the row (e.g. a landscape for the Visual stimulus) and its alternative (e.g. a wall for the Visual stimulus) minus the liking rating between vertical and horizontal lines. R values corresponds to Mann-Whitney U-test effect size and p values are statistics from a two-sided Wilcoxon signed-rank test comparing delta liking rating to zero, and to 50 for other measures.*

Post-learning session

To test whether the acquired reinforcing strength of the cues were long lasting, we added an additional block at the end of Experiment 1 and 2, where participants had to

select a cue between all possible combinations of cue pairs. For example, a participant could be shown the cue associated with 'information' next to the cue association with 'confirmation'. This resulted in 45 pairs, presented each four trials, resulting in 180 trials. The structure of the trial was as in the other blocks, without any feedback. Table S2 summarises the results. As can be seen, cues leading more often to rewards were chosen more than cues leading to the alternative. This suggests that the association between the reward and the abstract cue was durable above and beyond the learning sessions.

A

	L	I	C	M	V	W	S	D	N	H
Landscape (L)	-									
Information (I)	48.7±3.2	-								
Confirmation (C)	49.7±3.2	48.2±3.3	-							
Coin (M)	64.3±3.2**	63.1±3.1**	62.2±3.2***	-						
Vertical (V)	36.4±3.2**	35.4±3.1***	37.6±3.3***	28.4±2.9***	-					
Wall (W)	31.7±3.2***	33.6±3.2***	35.9±3.1***	26.8±3.0***	44.1±3.2	-				
String of letters (S)	30.2±3.1***	29.9±3.1***	32.2±3.1***	24.8±2.8***	44.6±3.2	43.8±3.1	-			
Disconfirmation (D)	32.9±3.0***	28.0±2.9***	31.7±3.1***	27.3±2.9***	44.3±3.2	50.2±3.2	54.5±3.2	-		
No coin (N)	32.7±2.9***	29.4±3.0***	28.7±2.9***	20.8±2.7***	39.6±3.2*	41.4±3.1	48.2±3.2	44.1±3.1	-	
Horizontal (H)	37.8±3.2**	36.7±3.1***	38.4±3.1**	30.7±3.0***	50.0±3.2	52.9±3.3	59.2±3.2*	56.2±3.2	64.3±3.1***	-

B

	L	I	C	M	V	W	S	D	N	H
Landscape (L)	-									
Information (I)	50.5±2.8	-								
Confirmation (C)	44.0±2.8	42.0±2.8**	-							
Coin (M)	60.8±2.8**	58.4±2.8**	65.4±2.8***	-						
Vertical (V)	35.6±2.8***	34.0±2.8***	39.8±2.8***	30.9±2.8***	-					
Wall (W)	29.7±2.8***	28.6±2.8***	33.4±2.8***	27.1±2.8***	41.0±2.8**	-				
String of letters (S)	26.9±2.8***	26.6±2.8***	32.4±2.8***	24.3±2.8***	39.2±2.8***	47.7±2.8	-			
Disconfirmation (D)	32.2±2.8***	28.9±2.8***	33.1±2.8***	26.9±2.8***	41.2±2.8**	52.8±2.8	54.1±2.8	-		
No coin (N)	27.3±2.8***	29.4±2.8***	29.7±2.8***	23.3±2.8***	36.0±2.8***	44.8±2.8	48.9±2.8	44.1±2.8	-	
Horizontal (H)	33.0±2.8***	29.5±2.8***	36.7±2.8***	26.5±2.8***	44.1±2.8	54.0±2.8	58.0±2.8*	52.8±2.8	58.4±2.8*	-

Table S4. Cues leading more often to rewarding stimuli are chosen more than cues leading to alternative stimuli in the post learning choice session for both Experiment 1 (A, N = 132 participants), 2 (B, N = 171 participants). *Letters on top of each column represent the stimulus explicitly named in the rows. Each number represents the percentage of time the cue associated with the row is chosen when presented with the cue associated with the column; for example, for the top left value, the cue associated with Information is chosen 50.5%±2.8 when presented with the cue associated with Landscape. Stars reflect p-values from two-sided Wilcoxon signed-rank test of each proportion against 50 (chance level). *p<0.05,**p<0.01, ***p<0.001*

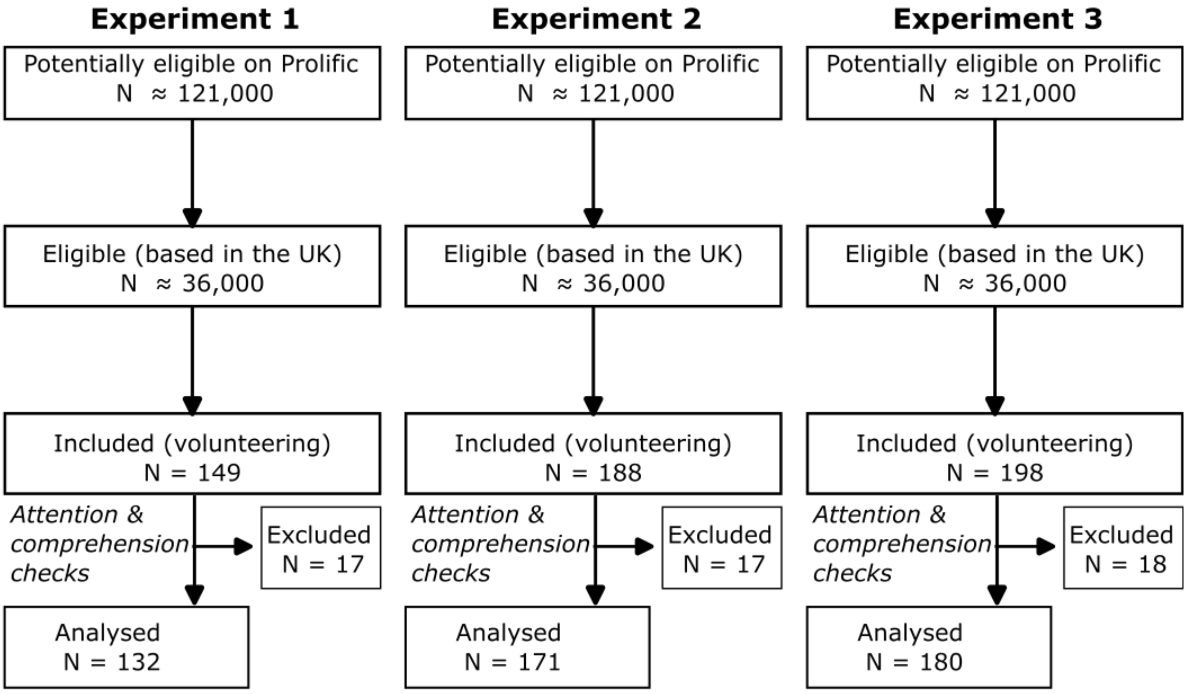

Figure S1. STROBE flow chart of the cross-sectional experiment. *We collected participants on the Prolific online platform, from which only participants based in UK were eligible (because we used pictures of coin in £ across all the experiments). Once an experiment is posted, eligible participants sign up and complete the study until all slots are taken. Participants who fail to meet the attention & comprehension checks criteria are excluded (see Methods, main text). Data of all other participants are analysed.*

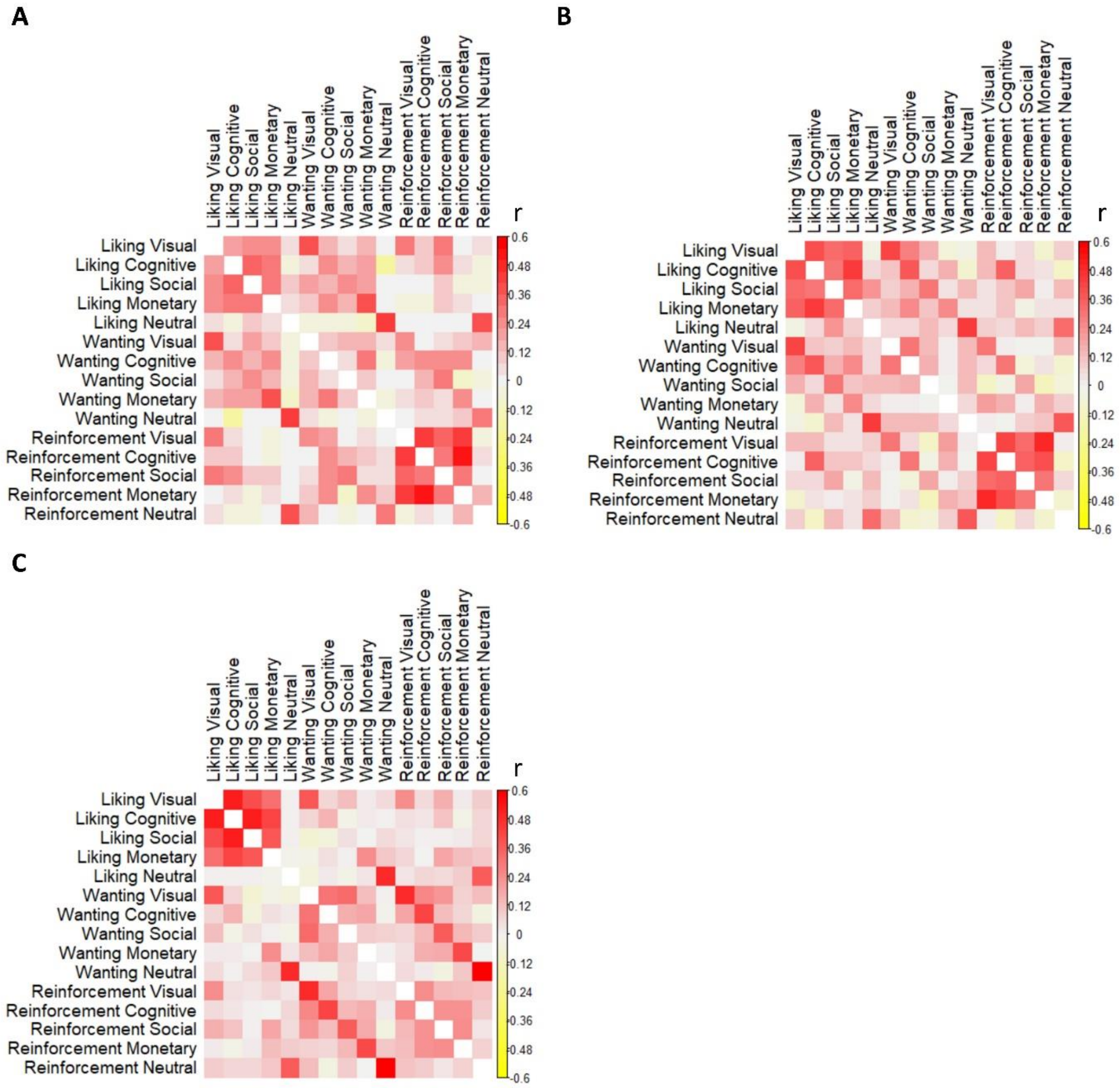

Figure S2. Correlation matrix across all measures (liking, wanting, reinforcing) and reward types for Experiment 1 (A, N = 132 participants), 2 (B, N = 171 participants), & 3 (C, N = 180 participants)3 (A-C). *Correlation strength "Pearson r" values (indicated by the colour intensity).*

Factors related to the Factor Analysis across all reward types.

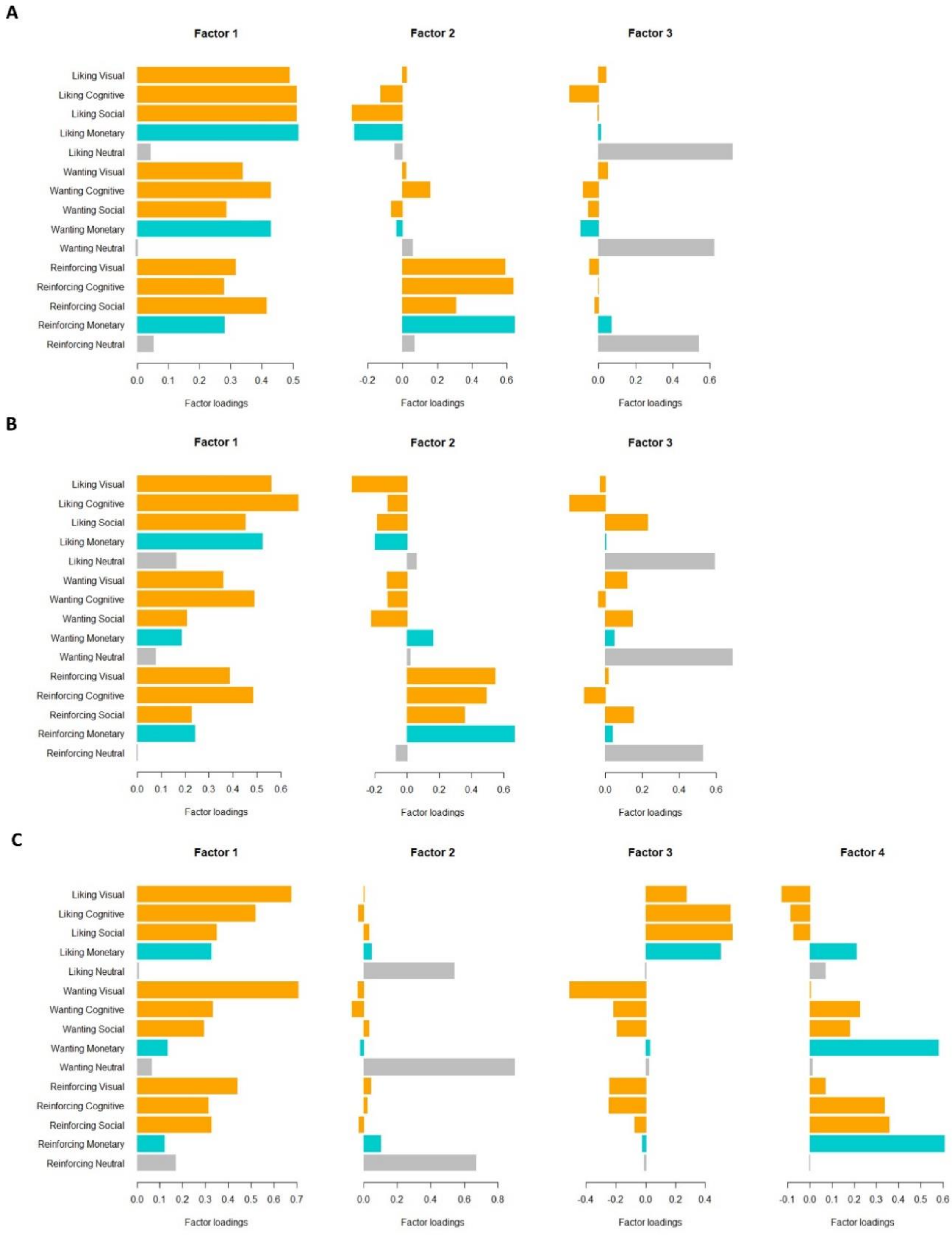

Figure S3. Factors' loadings in Experiment 1 (A, N = 132 participants), 2 (B, N = 171 participants), & 3 (C, N = 180 participants). *Factor 1 can be interpreted as a general reward sensitivity. Factor 2 in Experiment 1&2 and factor 3 in Experiment 3 can be interpreted as a response measure with positive loadings on choice measure and a negative loadings on the Likert rating. This factor likely captures noise inherent*

to these measures. Factor 3 in Experiment 1&2 and factor 2 in Experiment 3 corresponds to the sensitivity to the neutral stimuli. Factor 4 in Experiment 3 correspond mainly, but not solely, to monetary rewards.

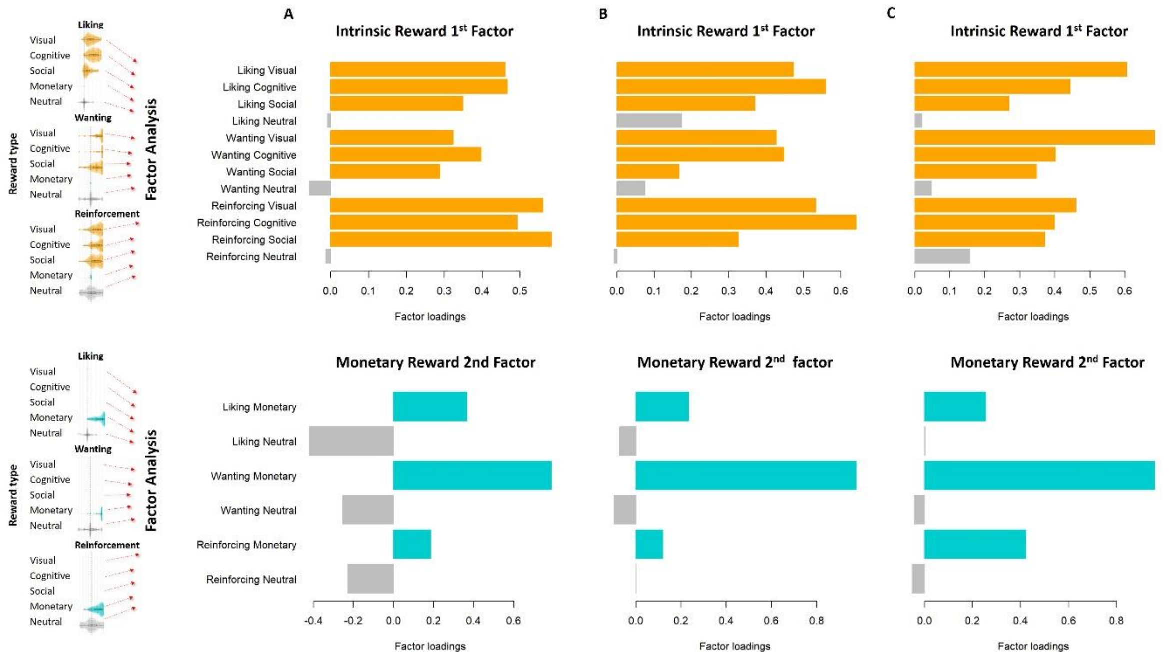

Figure S4. Factor Analysis separate for monetary reward and Intrinsic rewards in Experiment 1 (A, N = 132 participants), 2 (B, N = 171 participants), & 3 (C, N = 180 participants). *To assess whether the relationship between mental health and reward sensitivity was present for both intrinsic and monetary rewards separately, we performed two separate factor analyses: one including all three measures (liking, wanting, reinforcing) for intrinsic rewards (Visual, Cognitive, Social) and the neutral stimuli (top, orange & grey), and another including all three measures for monetary reward and neutral stimuli (bottom, blue & grey). The loadings were high for rewarding stimuli and low for neutral. The resulting score was then associated with the mental health score (see* ***Table 1****). Note that the violin plots on the left correspond to Figure 2 and are used to illustrate the data used in the factor analysis (see Figure 2 for details).*
